# Performance, Correlation and Kinetic Profile of Circulating Serum Fungal Biomarkers of Invasive Aspergillosis in High-Risk Patients with Hematologic Malignancies

**DOI:** 10.3390/jof7030211

**Published:** 2021-03-13

**Authors:** Maria Siopi, Stamatis Karakatsanis, Christoforos Roumpakis, Konstantinos Korantanis, Elina Eldeik, Helen Sambatakou, Nikolaos V. Sipsas, Panagiotis Tsirigotis, Maria Pagoni, Joseph Meletiadis

**Affiliations:** 1 Clinical Microbiology Laboratory, “Attikon” University General Hospital, Medical School, National and Kapodistrian University of Athens, 12462 Athens, Greece; marizasiopi@hotmail.com; 2 Unit of Bone Marrow Transplantation, Department of Hematology and Lymphoma, “Evangelismos” General Hospital, 10676 Athens, Greece; karakatsanisjstamatis@gmail.com (S.K.); marianpagoni@yahoo.com (M.P.); 3 Hematology Unit, 2nd Department of Internal Medicine, “Attikon” University General Hospital, Medical School, National and Kapodistrian University of Athens, 12462 Athens, Greece; chrisroubak@gmail.com (C.R.); panagtsirigotis@gmail.com (P.T.); 4 Pathophysiology Department, “Laiko” General Hospital, Medical School, National and Kapodistrian University of Athens, 11527 Athens, Greece; konstantinos_korinthos26@yahoo.gr (K.K.); nsipsas@med.uoa.gr (N.V.S.); 5 2nd Department of Internal Medicine, “Hippokration” General Hospital, 11527 Athens, Greece; elinaeldik@yahoo.gr (E.E.); helensambatakou@msn.com (H.S.)

**Keywords:** invasive aspergillosis, hematology patients, diagnosis, biomarkers, kinetic profile

## Abstract

As conventional microbiological documentation of invasive aspergillosis (IA) is difficult to obtain, serum fungal biomarkers are important adjunctive diagnostic tools. Positivity rates and the kinetic profiles of galactomannan (GM), 1,3-β-D-glucan (BDG) and *Aspergillus* DNA (PCR) were studied in high-risk patients with hematologic malignancies. GM, BDG and PCR data from serial serum specimens (*n* = 240) from 93 adult hematology patients with probable (*n* = 8), possible (*n* = 25) and no (*n* = 60) IA were retrospectively analyzed. Positivity rates and sensitivity/specificity/positive/negative predictive values (NPV) of each fungal biomarker alone and in combination were estimated. The three markers were compared head-to-head and correlated with various biochemical, demographic and patient characteristics. The positivity rates for patients with probable/possible/no IA were 88%/8%/0% for GM (X^2^ = 55, *p* < 0.001), 62%/46%/35% for BDG (X^2^ = 2.5, *p* = 0.29), 62%/33%/27% for PCR (X^2^ = 3.9, *p* = 0.15), 50%/4%/0% for GM + BDG and GM + PCR (X^2^ = 31, *p* < 0.001), 50%/8%/22% for BDG + PCR (X^2^ = 6.5, *p* = 0.038) and 38%/4%/0% for GM + BDG + PCR (X^2^ = 21, *p* < 0.001). Higher agreement (76%) and negative correlation (r_s_ = −0.47, *p* = 0.0017) was found between GM index and PCR Ct values. The sensitivity and NPV was 45–55% and 90–92% when biomarkers assessed alone and increased to 75–90% and 93–97%, respectively when combined. Weak significant correlations were found between GM, PCR and BDG results with renal/liver function markers (r = 0.11–0.57) with most GM+ and PCR+ samples found in the first and second week of clinical assessment, respectively and BDG later on. Different positivity rates, time profiles and performances were found for the three biomarkers advocating the combination of GM with PCR for the early diagnosis of IA, whereas the high NPV of combined biomarkerscould help excluding IA.

## 1. Introduction

Invasive aspergillosis (IA) continues to pose a challenge in the management of hematology patients. Delayed initiation of targeted therapy increases mortality, making early and accurate diagnosis vital for a successful clinical outcome [1]. Although conventional microbiological and radiological techniques are considered the cornerstone of IA diagnosis, they are not sufficiently sensitive and highly specific, respectively [2], and their performance are confounded by several factors [3,4,5]. *Aspergillus* galactomannan (GM) antigen is the biomarker that is most often used in current practice to diagnose IA, guide the early administration of antifungal therapy and monitor response to treatment. 1,3-β-D glucan (BDG) antigenemia has been incorporated in the definition of a probable invasive fungal disease [6], although BDG is a pan-fungal marker and is not specific for *Aspergillus* spp. Recently, for the first time, polymerase chain reaction (PCR)-based assays were included as mycological evidence to help define episodes of probable IA [6]. 

To date, multiple studies have highlighted the utility of serial testing of different serum IA biomarkers in predicting clinical response outcome and survival in hematology patients [7,8]. Nevertheless, the kinetics of each of these biomarkers and the impact of various patients’ characteristics on them and on the diagnosis of IA has not been investigated thoroughly [9]. Preclinical studies demonstrated different kinetics of these biomarkers in vitro and in vivo in animal models [10,11]. As these biomarkers are eliminated via the kidney, liver or neutrophils, the diagnostic performance of these assays may be influenced by renal and hepatic function and other patients’ characteristics as described in liver transplant recipients [12]. Given the complex clinical picture of IA, a more dynamic approach for the evaluation of diagnostic biomarkers is warranted in order to understand the kinetics of each biomarker, confounding factors and diagnostic performance. Therefore, the aim of the present retrospective study was to evaluate the performance of the detection of circulating serum fungal biomarkers of IA, GM, BDG and *Aspergillus* DNA, in serum samples of high-risk patients with hematologic malignancies with a focus on describing their kinetic profile, identifying significant correlates and combining them in order to increase diagnostic performance. 

## 2. Materials and Methods

### 2.1. Study Design and Population

A total of 93 adult patients with hematologic malignancies at risk for IA [13,14,15] according to the attending clinicians (patients with anticipated prolonged and profound neutropenia) were screened for the detection of GM, BDG and *Aspergillus* DNA in serum samples collected during a 6-month period in each of four tertiary care hospitals in the area of Athens, Greece, namely “Attikon” University General Hospital (*n* = 21), “Evangelismos” General Hospital (*n* = 39), “Hippokration” General Hospital (*n* = 12) and “Laiko” General Hospital (*n* = 21). Patient episodes (proven, probable, possible or no evidence of IA) were stratified according to the 2020 definitions of the European Organization for Research and Treatment of Cancer-Invasive Fungal Infections Cooperative Group/National Institute of Allergy and Infectious Diseases Mycosis Study Group Education and Research Consortium (EORTC/MSGERC) Consensus Group [6]. Patients’ demographic (gender, age, underlying disease) and clinical characteristics during the survey period (duration and degree of neutropenia (absolute neutrophil count (ANC) <500/mm^3^), hepatic and renal function, antifungal treatment and severity scores (Child-Pugh, SAPSII, APACHEIII and Glasgow Comma scores) were obtained from computerized databases of each center. 

The study protocol was approved by the local institutional Review Board and Bioethics Committee of each participating hospital and written informed consent was obtained from each patient or relative. 

### 2.2. Clinical Samples and Biomarker Testing

Serial serum specimens from all patients were collected. The number of evaluable serum samples for the detection of fungal biomarkers was 240. For most patients there were several samples (3–15) during neutropenia. The obtained sera were stored at −70 °C until testing. A commercially available sandwich enzyme-linked immunoassay (Platelia *Aspergillus* EIA; Bio-Rad Laboratories, Athens, Greece) was used to quantify GM antigen in accordance with the manufacturer’s instructions. A result was considered positive when index value was ≥0.5 [16]. For IA classification based on 2020 EORTC/MSGERC criteria a GM index ≥1 was used [6]. BDG was detected with the Fungitell^®^ test kit (Associates of Cape Cod, Inc., Falmouth, MA, USA), as recommended by the manufacturer. BDG levels of ≥80, 79–60 and <60 pg/mL were considered positive, indeterminate and negative, respectively. Serum assays were performed in duplicate [17]. A real-time PCR was developed in line with the published European *Aspergillus* PCR Initiative recommendations for serum [18]. *Aspergillus* DNA was extracted from 1 mL serum after enzymatic (incubation with protease K at 56 °C for 10 min) and mechanical (15 min vortex with glass beads) pre-treatment using the High Pure Viral Nucleic Acid Large Volume Kit (Roche, Athens, Greece) according to the manufacturer’s instructions. Real-time PCR was performed with a previously validated assay (2Asp assay) using *Aspergillus*-specific primers (ASF1 and ADR1) targeting the 28S rRNA gene and an Aspergillus-specific hydrolysis probe (ASP28P) [19,20]. All PCR runs included a positive control and a negative control (water in place of DNA extract). When no amplification was observed after 43 PCR cycles (C_t_), the sample was considered negative by PCR [20]. The mean ± SD Cts of spiked human sera with 10, 100 and 1000 CFUs were 27.8 ± 1.1, 33.5 ± 1.1 and 39.2 ± 0.7, respectively from different runs which are in agreement with previous studies [19,20]. 

### 2.3. Data Analysis

(i) Descriptive statistics. Median and interquartile ranges (IQR) were calculated for continuous variables, while numbers and percentages were calculated for categorical parameters. (ii) Analytical evaluation. GM indices, BDG concentrations and PCR C_t_ values were correlated with each other and with patient demographics, biochemical parameters and white blood cells (WBCs) using Spearman correlation, ANOVA analysis and linear regression. Categorical variables like GM, BDG and PCR positivity were compared with qualitative categories (antifungal prophylaxis, acute myelogenous leukemia (AML), autologous hematopoietic stem cell transplantation (HSCT), neutropenia) using chi-square and Fisher’s exact test. (iii) Diagnostic evaluation. The three fungal biomarkers were compared head-to-head and conclusions about their performance were made. Indeterminate BDG values were considered as negative for comparison with the other biomarkers. Sensitivity/specificity rates and positive/negative predictive values (PPV/NPV) of BDG and PCR alone and in combination were assessed using two-by-two tables and the 95% confidence interval (CI) was estimated. The area and the *p* value of receiver-operating characteristics (ROC) curves were calculated for PCR and BDG for patients with probable vs. no IA, probable + possible IA vs. no IA and probable vs. possible + no IA. (iv) Time-to-positivity. The median (range and IQR) days after clinical assessment that samples were positive for each of three biomarkers were calculated and the relative time compared to the time-to-positivity of each biomarker were determined for patients with probable IA. The cumulative probability distributions of each biomarker for patients with probable IA over time was constructed and compared. A two-tailed *p*-value of <0.05 was considered to reveal a statistically significant difference. All data were analyzed using the statistics software package GraphPad Prism, version 7.0, for Windows (GraphPad Software, San Diego, CA, USA) and JMP7 software (SAS Institute, Cary, NC, USA).

## 3. Results

### 3.1. Patients’ Characteristics and IA Episodes

Of 93 patients enrolled in the study, 48/93 (52%) were men with median (range, IQR) age 51 (18–83, 27) years, weight 69 (48–115, 17) kg, height 168 (145–183, 11) cm, body mass index (BMI) 24 (14–37, 5) kg/m^2^, WBCs 0.46 (0–37.14, 1.11) × 10^9^/mL (91% with neutropenia, 76% with grade IV), SAPSII score 31 (16–78, 7), APACHEIII score 13 (6–35, 5), Glasgow Comma score 15 (3–15, 0), Child-Pugh 6 (5–10, 1), creatinine 0.6 (0.24–3.2, 0.4) mg/dL, eGFR 118 (16–386, 86) mL/min/1.73 m^2^, urea 28 (0.5–132, 20) mg/dL, SGOT 18 (5–306, 15) U/L, SGPT 25 (2–221, 30) U/L, γ-GT 48 (0.66–1098, 59) U/L, bilirubin 0.63 (0.14–5.6, 0.44) mg/dL and ALP 75 (11–562, 56) U/L. The most common underlying hematological disorder was AML (62/93; 67%), followed by acute lymphoblastic leukemia (12/93; 13%), myelodysplastic syndrome (5/93; 5%), non-Hodgkin’s lymphoma (2/93; 2%) and various other conditions, such as myeloma, chronic lymphocytic leukemia, chronic myeloid leukemia, Burkitt lymphoma and Hodgkin disease (12/93; 13%). Among patients, 22/93 (24%) had undergone autologous HSCT.

There were 8 (9%) patients with probable IA, 25 (27%) cases classified as possible IA and 60 (64%) with no IA, while no proven IA was documented. Most of the patients (76/93; 82%) had received ≥2 defined daily doses of antifungal drugs, with 40% being on mold-active prophylaxis/treatment (19 voriconazole, 5 posaconazole, 3 itraconazole, 3 liposomal amphotericin B) at the time serum samples were collected. 

### 3.2. Correlation between Fungal Biomarkers and Various Parameters

**Figure 1:**
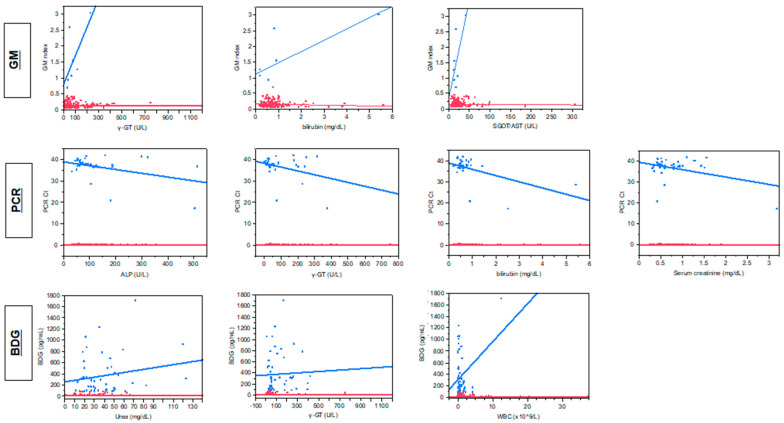
Correlation between fungal biomarkers [galactomannan (GM), 1,3-β-D glucan (BDG) and *Aspergillus* DNA (PCR] and markers of hepatic (γ-GT, bilirubin, SGOT/AST, ALP) and renal function (serum creatinine, urea) and white blood cell counts for positive (blue lines) and negative (red lines) samples.

Quantitative correlation of the levels of the three fungal biomarkers with different biochemical parameters indicates a significant correlation only between BDG concentrations and urea (r_s_ = 0.18, *p* = 0.02) and γ-GT (r_s_ = 0.18, *p* = 0.02). When only samples positive of each biomarker were analyzed separately with linear regression, significant correlation was found between PCR C_t_ and γ-GT (r = 0.12, *p* = 0.039), bilirubin (r = 0.28, *p* = 0.011), ALP (r = 0.17, *p* = 0.014), creatinine (r = 0.15, *p* = 0.023) and borderline significant correlation between GM index and γ-GT (r = 0.47, *p* = 0.08), SGOT (r = 0.48, *p* = 0.08), bilirubin (r = 0.57, *p* = 0.049) and between BDG concentrations and WBCs (r = 0.11, *p* = 0.014) (Figure 1). BDG positivity was higher for patients not on antifungal treatment vs. those on antifungal treatment (68% vs. 33%, *p* = 0.0007) with the mean ± SEM 354 ± 62 vs. 143 ± 22 pg/mL (*p* = 0.0016). 

### 3.3. Biomarkers per Sample

**Figure 2:**
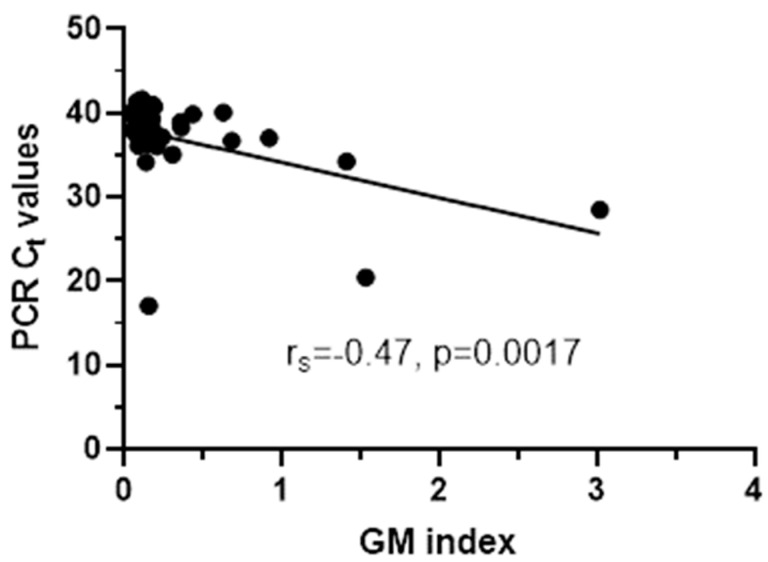
Significant correlation between PCR C_t_ values and GM indices for PCR+ samples.

Out of 240 serum samples tested for GM, 11 (5%) were positive with a median (range, IQR) GM index value of 1.05 (0.51–3.02, 0.76). Concerning the BDG testing, 81 (34%) samples were positive with a median (range, IQR) BDG concentration of 309 (84–1719, 430) pg/mL, 7 (3%) were indeterminate and 152 were negative. For *Aspergillus* real-time PCR there was sufficient volume for testing 156/240 (65%) samples, whereof 39 (25%) were positive with a median (range, IQR) C_t_ 37.7 (17.1–41.6, 2.6). Thus, taking into account only the 156 samples tested for all three biomarkers, 88 (56%) were positive in at least one biomarker, whereas only 4 (3%) were positive in all three (Table 1). The four samples were from four patients (two AML, all on antifungal treatment, three with antimold therapy), three with probable IA and one with possible IA and had GM index/PCR C_t_/BDG pg/mL 1.41/34/119, 0.63/40.1/320, 3.02/28.47/309, 1.53/20.45/84, respectively. The sample positivity rate of BDG was higher in patients with probable (55%) and possible IA (46%) than in patients with no evidence for IA (40%), whereas the positivity rate of PCR was higher in patients with probable IA (55%) than in patients with possible IA (19%) and no evidence for IA (22%). When samples positive to one of the biomarkers were analyzed, significant correlation was found between PCR C_t_ and GM indices (r_s_ = −0.47, *p* = 0.0017) (Figure 2).

### 3.4. Biomarkers per Patient

Biomarkers per patient were analyzed for the 156 serum samples from 83/93 (89%) patients where data for all three biomarkers were available (Table 1). Of 83 patients, 45 (54%) were positive in at least one biomarker. In particular, 9 (11%), 35 (42%) and 27 (33%) were GM, BDG and PCR positive, respectively, in at least one sample, while 2 (2%), 22 (26%) and 9 (11%) were GM, BDG and PCR positive, respectively, in at least two samples. No patients had more than two, four and three consecutive samples positive for GM, BDG and *Aspergillus* DNA, respectively. Only 4/83 (5%) patients were positive in all three assays, although 38% of patients with probable IA were positive in all three biomarkers. Differences in positivity rates between patients with probable (62%) or possible IA (46%) and patients with no IA (35%) for BDG and between patients with probable IA (62%) and patients with possible IA (33%) and no IA (27%) for PCR was greater when analysis was performed per patient (i.e., any sample from the same patient being positive). Significant association between 2020 IA classification was found only for GM (*p* < 0.0001) and PCR (*p* = 0.0013) but not for BDG (*p* = 0.83). The positivity rates for patients with probable/possible/no IA were 88%/8%/0 % for GM (X^2^ = 55, *p* < 0.001), 62%/46%/35% for BDG (X^2^ = 2.5, *p* = 0.29), 62%/33%/27% for PCR (X^2^ = 3.9, *p* = 0.15), 50%/4%/0% for GM + BDG and GM + PCR (X^2^ = 31, *p* < 0.001), 50%/8%/22% for BDG + PCR (X^2^ = 6.5, *p* = 0.038) and 38%/4%/0% for GM + BDG + PCR (X^2^ = 21, *p* < 0.001).

**Figure 3:**
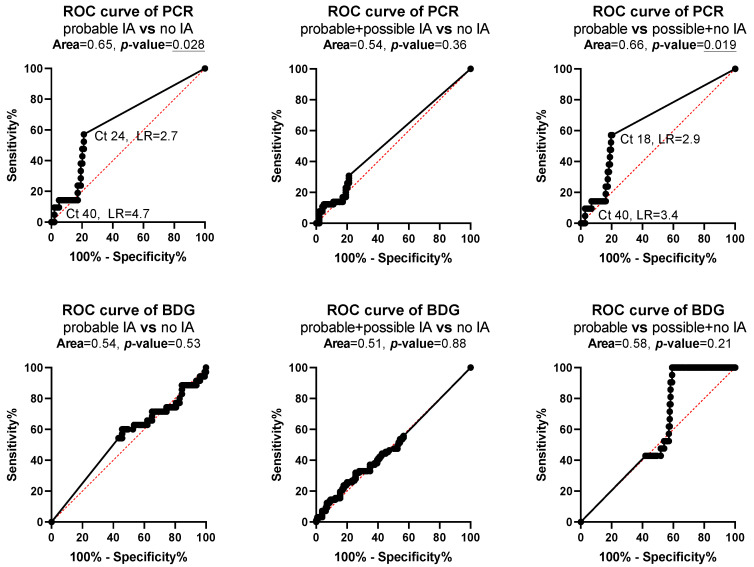
Receiver operating characteristic (ROC) curve analysis for polymerase chain reaction (PCR) and 1,3-β-D glucan (BDG) for patients with probable, possible and no invasive aspergillosis (IA). *p* values <0.05 are underlined indicating the test discriminate patients to different classifications of IA.

ROC curve analysis found statistically significant results for PCR when patients with probable IA were compared with patients with no IA (area = 0.65, *p* = 0.028) and possible IA (area = 0.66, *p* = 0.019) with the best specificity of 97% (93–99%) found at C_t_ 40 and the best sensitivity of 57% (37–76%) at C_t_ 18–24 (Figure 3).

### 3.5. Agreement between Biomarkers and Diagnostic Performance

The agreement between the GM-PCR, GM-BDG and PCR-BDG assays was 76%, 57% and 60%, respectively. The test characteristics for differentiating patients with probable IA, possible IA and no evidence for IA are shown in Table 2. Overall, higher NPV was found when patients with possible IA and no IA were combined and higher PPV when patients with probable and possible IA were combined. Moderate sensitivity (45–55%) and high NPV (90–92%) in differentiating patients with probable IA vs possible+no IA was found for GM, BDG and PCR alone whereas higher specificity (98%) and PPV (82%) was found for GM compared to PCR (79% and 28%, respectively) and BDG (58% and 16%, respectively). When biomarkers were combined (positive test to either one) sensitivity (75–90%) and NPV (93–97%) increased whereas specificity (48–79%) and PPV (18–36%) decreased. 

### 3.6. Time-To-Positivity

**Figure 4:**
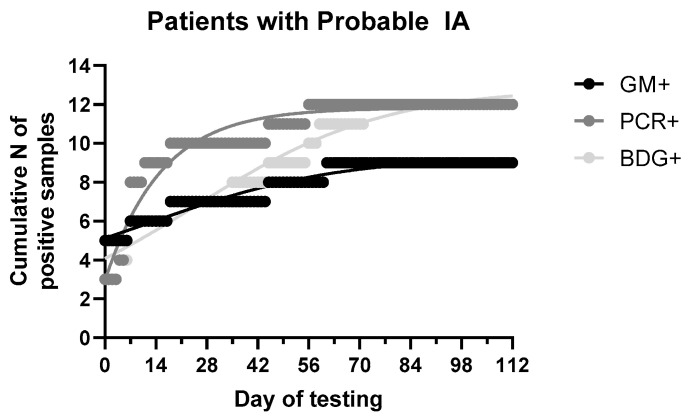
Cumulative distribution of fungal biomarkers [galactomannan (GM), 1,3-β-D glucan (BDG) and *Aspergillus* DNA (PCR)] in patients with probable invasive aspergillosis (IA).

The median (range, IQR) time of positive results among patients with probable IA was 0 (0–61, 18), 7 (0–56, 10) and 18 (0–71, 45) days of testing when samples were collected based on clinical assessment for GM, PCR and BDG (Figure 4). As detailed in Table 1, GM was positive in 7/8 patients with probable IA (the eighth patient had PCR+ and GM- with GM indices 0.09–0.25), while BDG and PCR in 5/8. Of eight patients with probable IA, only one had two GM+ samples and none in consecutive samples, whereas 3/5 PCR+ patients had consecutive PCR+ samples and 2/5 BDG+ patients had consecutive BDG+ samples. Comparing the time-to-positive result for each assay, for the four GM+/PCR+ patients with probable IA, GM and PCR were positive at the same day for two patients, whereas for the other two patients GM precedes PCR by 4 days in the first patient and PCR precede GM by 38 days for the second patient. For the four GM+/BDG+ patients with probable IA, GM and BDG were positive at the same day for two patients whereas for the other two patients GM precedes BDG by 18 days for the first patient and BDG precedes GM by 10 days for the second patient. Finally, for the four PCR+/BDG+ patients with probable IA, PCR and BDG were positive at the same day for one patient, whereas PCR precedes BDG by 18 and 28 days in two patients and BDG precedes PCR by 4 days in one patient. Analysis of cumulative probabilities indicates that more samples were relatively tested GM+ compared to PCR and BDG in the first week of testing whereas in the second week more samples were tested PCR+ compared to GM and BDG (Figure 4). After 6 weeks BDG+ samples were relatively more than GM+ approaching the number of PCR+ samples after 10 weeks. Most (80%) positive samples were found within first 2, 4 and 8 weeks with PCR, GM and BDG, respectively. 

## 4. Discussion

Comparison between fungal biomarkers and understanding their kinetics may have a significant impact on their diagnostic performance. In the present retrospective multicenter study, among the three biomarkers, the highest agreement (76%) was found between GM and PCR which is supported by the significant negative correlation between GM indices and PCR C_t_ values (samples with high GM indices had more fungal DNA). BDG and PCR positivity rates were higher in patients with probable IA than in patients with possible IA or no IA. The sensitivity and NPV was 45–55% and 90–92% when biomarkers assessed alone and increased to 75–90% and 93–97%, respectively when combined. ROC curve analysis for PCR showed that the highest sensitivity (57%) and specificity (97%) for probable IA was found for C_t_ 18–24 and 40, respectively. A total of 54% of patients were positive in at least one biomarker whereas, based on all three assays, the positivity rate among high-risk patients was only 5%. Biomarkers were positive until 2 months after clinical suspicion of IA with more GM+ and PCR+ samples found in the first and second week, respectively, whereas most BDG+ samples become positive in later ones. Thus, the combination of GM and PCR would capture most patients with probable IA the first 2 weeks after clinical suspicion of IA, whereas the higher NPV of BDG and/or PCR could be used to exclude IA. Significant but weak correlation was found between GM, PCR and BDG results with renal/hepatic function markers whereas BDG results were also associated with antifungal treatment.

The limitations of the traditional diagnostic techniques for IA have led to the development of non-culture tests based mainly on the detection of antigens or nucleic acids of the genus *Aspergillus*. GM is a major component of the *Aspergillus* cell wall and the determination of its antigen in serum and bronchoalveolar lavage specimens has been endorsed as a standard non-invasive tool for the diagnosis of probable IA [20]. In a meta-analysis of 27 studies, the mean (95% CI) sensitivity and specificity of GM assay in serum of patients with proven or probable IA was 69% (59–79%) and 89% (84–94%), respectively. However, remarkable variability was observed when subgroup analysis of proven cases was stratified by the underlying disease. In particular, the mean (95% CI) sensitivity/specificity of the test for hematology patients, bone marrow transplant recipients and recipients of solid-organ transplants were 70% (62–77%)/92% (90–93%), 82% (70–90%)/86% (83–88%) and 22% (3–60%)/84% (78–88%), respectively [21]. In addition, the administration of antifungal therapy reduces the sensitivity of the test to 20%. A low sensitivity was also found in present study (45%) in differentiating patients with probable IA vs possible+no IA because of extended use of antifungal therapy. Nevertheless, its greatest value lies in the serial screening of patients with hematologic malignancies at high risk for IA, like AML undergoing intensive chemotherapy, demonstrating 92% sensitivity and 98% specificity when two consecutive serum samples are positive [22]. High specificity (98%) of GM in differentiating patients with probable IA vs possible+no IA was also found in the present study. 

The quantification of BDG, a cell wall polysaccharide found in almost all pathogenic fungi, can be used as a pan-fungal marker. A meta-analysis showed that the sensitivity of BDG testing for patients with proven/probable IA ranged from 60 to 100% [23]. In general, BDG detection helps to exclude *Aspergillus* infections (NPV > 90%), but BDG is not an IA-specific marker and false positive results have been related to several factors [24,25]. Given the low sensitivity (55%) and PPV (16%) in differentiating patients with probable IA vs possible+no IA, the assay’s contribution in the diagnosis of IA in our population was modest, which is in agreement with previous findings suggesting BDG’s limited usefulness as a screening method for invasive fungal infections in hematology patients [26]. In the present study, although the positivity rate was higher in patients with IA than in patients without IA and improved when multiple samples per patient were analyzed, the positivity rate was the same between patients with probable and possible IA. However, BDG was not correlated with 2020 classification criteria supporting the exclusion of this biomarker for the diagnosis of IA [6]. Nevertheless, its excellent NPV (90%) could probably indicate a potential role in excluding invasive fungal infections including IA. 

On the other hand, molecular diagnosis of IA encounters obstacles in its broad acceptance due to lack of standardization with regard to the most appropriate sample, DNA extraction and PCR reaction. This has led to variable performances of individual *Aspergillus* PCR protocols, with sensitivities and specificities ranging from 36 to 100% and from 80 to 96%, respectively [22], while a recent meta-analysis showed that the pooled diagnostic performance of whole-blood and serum PCR assays was moderate, with mean (95% CI) sensitivity and specificity of 84% (75–91%) and 76% (65–84%), respectively [27], which is in line with the specificity found in the present study (9%) in differentiating patients with probable IA vs possible+no IA. The sensitivity was slightly lower in the present study (55%) probably because a dense sampling schedule (strictly twice a week) was not followed. However, the positivity rate of PCR in patients with probable IA (62%) was higher than in patients with possible (33%) or no IA (27%). ROC curves indicate that samples with C_t_ > 40 were found more frequently in patients without IA which is line with the C_t_ of 43 proposed for PCR negative samples [20]. The relatively hig C_t_ among patietns with probable IA indicate low circulating serum fungal DNA. Given this heterogeneity, *Aspergillus* PCR has been proposed as a screening test in conjunction with other diagnostic markers to ensure maximum sensitivity and NPV [28,29]. As with BDG, its excellent NPV (92%) demonstrated in our study as well as in previous reports [30] may suggest its benefit for excluding IA.

Given the challenges in the diagnostic process of IA, many studies have recommended the application of combined biomarker screening in high-risk hematology patients [31,32]. Only 5% of patients (38% of probable IA cases) were positive for all three serum biomarkers tested at the same time point. It is well known that pathogens possess a rich metabolism and a variety of secondary metabolites, many unique to their species. In fact, each metabolite is detected in different stages of IA and with a different duration, as evidenced by the moderate levels of agreement (57–76%) and time-to-positivity profiles of the three biomarkers tested in this study. When fungal biomarkers were combined, the sensitivity and NPV increased reaching 90% and 97%, respectively when all three biomarkers were combined whereas specificity and PPV decreased compared to GM alone. Therefore, combination of diagnostic tests may increase the NPV whereas GM alone results in high PPV. 

Several factors may affect the levels of the three fungal biomarkers in serum and thereby their diagnostic performance. Although weak, significant correlations with consistent pattern was found for each of the three biomarkers with several markers of renal and hepatic function, patient group and antifungal treatment. Polysaccharides [33] and microbial DNA [34,35] are metabolized in liver and therefore any changes in liver function may affect their levels. GM is excreted in urine [36,37] accounting for 35% of the radiolabeled GM injected in rabbits after 24 h [33]. Similarly BDG has been detected in urine from patients with IA [38]. Hence, renal and hepatic dysfunction can alter elimination of those biomarkers and probably increase their detection which is in line with the positive correlation found between GM indices and liver enzymes. Correlation between GM index and renal function markers was non-significant which may indicate a minor role of GM renal elimination or low power in detecting such a correlation. Regarding BDG, a positive correlation was found with renal and hepatic function markers. Whether the correlation between BDG with urea is related with previous observations of protective role of BG against nephropathy in animals due to antioxidant and immunomodulatory [39] or urinary excretion of (1,3)b-d-glucans [40] needs to be explored further. Finally, PCR C_t_ values were negatively correlated with renal and hepatic function markers (i.e., more DNA copies were detected in patients with renal and hepatic dysfunction) which may be due to significant quantitative relation found with severity scores. Unfortunately, the diagnostic impact of these confounding variables could not be addressed in the present study because larger number of positive samples and wider distribution of confounding variables will be required. In addition, the small samples size and particularly for patients with probable IA, the absence of proven IA and the differences among centers and patients may have an impact on observed findings in the present study.

In conclusion, diagnosis of IA remains challenging. GM can help in the diagnosis of IA, whereas serial sampling improves significantly the positivity rate of PCR in patients with probable IA. Combination of BDG with GM or PCR did not increase significantly the diagnostic performance. The absence of highly sensitive and specific diagnostic indicators impedes the successful clinical outcome of IA, while none of the current assays (serological or molecular) alone are able to confirm the infection and their results should always be evaluated in conjunction with other clinical, radiological and microbiological findings. However, the high NPV of those assays particularly when serial sampling is employed can help to exclude IA with major implications in prophylactic and de-escalation strategies. Renal/hepatic function and other factors (neutropenia, patient group, treatment) that may influence fungal biomarkers levels should be considered. Understanding the kinetics of each biomarker and influencing variables can help to optimize diagnostic performance of serological and molecular diagnostic tools of IA. 

## Figures and Tables

**Table 1:** Positivity rates of galactomannan (GM), 1,3-β-D glucan (BDG) and *Aspergillus* polymerase chain reaction (PCR) assays in patients with probable, possible and no invasive aspergillosis (IA) with respective to the European Organization for Research and Treatment of Cancer-Invasive Fungal Infections Cooperative Group/National Institute of Allergy and Infectious Diseases Mycosis Study Group Education and Research Consortium (EORTC/MSGERC) 2020 classification of patients [6].

Patient Classification	(No of Samples/Patients)	No of Samples/Patients Positive for Indicated Assay						
		GM	BDG	PCR	GM + BDG	GM + PCR	BDG + PCR	GM + BDG + PCR
**Probable IA**	Samples (*n* = 20)	9 (45%)	11 (55%)	11 (55%)	5 (25%)	5 (25%)	7 (35%)	3 (15%)
	Patients (*n* = 8)	7 (88%)	5 (62%)	5 (62%)	4 (50%)	4 (50%)	4 (50%)	3 (38%)
**Possible IA**	Samples (*n* = 43)	2 (5%)	20 (46%)	8 (19%)	1 (2%)	1 (2%)	2 (5%)	1 (2%)
	Patients (*n* = 24)	2 (8%)	11 (46%)	8 (33%)	1 (4%)	1 (4%)	2 (8%)	1 (4%)
**No evidence for IA**	Samples (*n* = 93)	0 (0%)	37 (40%)	20 (22%)	0 (0%)	0 (0%)	13 (14%)	0 (0%)
	Patients (*n* = 51)	0 (0%)	18 (35%)	14 (27%)	0 (0%)	0 (0%)	11 (22%)	0 (0%)
**Total**	Samples (*n* = 156)	11 (7%)	68 (44%)	39 (25%)	6 (4%)	6 (4%)	22 (14%)	4 (3%)
	Patients (*n* = 83)	9 (11%)	34 (41%)	27 (32%)	5 (6%)	5 (6%)	17 (20%)	4 (5%)

**Table 2:** Test characteristics (sensitivity/specificity/positive predictive value/negative predictive value), of each fungal biomarker alone and in combination for differentiating patients with probable IA, possible IA and no evidence for IA.

Fungal Biomarkers	Probable IA vs. no IA	Probable+Possible IA vs. no IA	Probable IA vs. Possible+no IA
GM	45%/100%/100%/64%	17%/100%/100%/64%	45%/98%/82%/92%
PCR	55%/78%/35%/89%	30%/78%/49%/62%	55%/79%/28%/92%
BDG	55%/60%/23%/86%	49%/60%/46%/64%	55%/58%/16%/90%
PCR/BDG	75%/53%/25%/91%	65%/53%/48%/69%	75%/48%/18%/93%
PCR/GM	80%/78%/44%/95%	40%/78%/56%/66%	80%/79%/36%/96%
BDG/GM	80%/60%/30%/93%	59%/60%/50%/68%	80%/57%/22%/95%
PCR/BDG/GM	90%/53%/29%/96%	71%/53%/51%/73%	90%/48%/20%/97%

GM: galactomannan, BDG: 1,3-β-D glucan, PCR: polymerase chain reaction.

## Data Availability

Data available on request.
